# Identification of Differentially Expressed microRNAs between the Fenpropathrin Resistant and Susceptible Strains in *Tetranychus cinnabarinus*

**DOI:** 10.1371/journal.pone.0152924

**Published:** 2016-04-06

**Authors:** Yichao Zhang, Zhifeng Xu, Qiong Wu, Miao Peng, Yanchao Liu, Xing Liu, Li Shi, Guangmao Shen, Yu Pan, Lin He

**Affiliations:** 1 Key Laboratory of Entomology and Pest Control Engineering, College of Plant Protection, Southwest University, Chongqing, China; 2 College of Horticulture and Landscape Agriculture, Southwest University, Chongqing, China; Kunming University of Science and Technology, CHINA

## Abstract

The carmine spider mite (*Tetranychus cinnabarinus*) is one of the most serious pests on crops and its control mainly depends on chemical acaricides. The excessive and improper acaricides use has resulted in mite resistance to many acaricides, including fenpropathrin. Previous studies have indicated fenpropathrin resistance is a complex development process involving many genes, but information on resistance mechanism of post-transcription regulation is rare. Using Illumina sequencing, several categories of sRNAs were identified from susceptible (TS) and fenpropathrin-resistant strains (TR) of *T*. *cinnabarinus*, including 75 known microRNAs (miRNAs) and 64 novel miRNAs, whose target genes containing 78592 miRNA-target pairs were predicted by 6 algorithms. Also, 12 significantly differently expressed miRNAs were identified between the TS and TR libraries and RT-qPCR validation also performed a well consistency with sequencing. The targets of significantly differentially expressed miRNAs included 7 glutathione S-transferase, 7 cytochrome P450 and 16 carboxyl/choline esterase genes, their function in fenpropathrin resistance were further analyzed. The present study provides the firstly large-scale characterization of miRNAs in *T*. *cinnabarinus* and the comparison between TS and TR strains gives a clue on how miRNA involves in fenpropathrin resistance.

## Introduction

Not very long ago, the non-coding regions in the genomes of living organisms were considered to be junk DNA. In the last two decades, advances in molecular biology indicated that these regions of genomes can express non-coding RNAs (NcRNAs) which play significant roles in various aspects of cell and organismal biology [1]. Small RNAs (sRNAs) are less than 200 nucleotides (nt) long non-coding RNA molecules and classified into microRNAs (miRNAs), small nuclear RNAs (snRNAs), small interfering RNAs (siRNAs), piwi-associated RNAs (piRNAs), small nucleolar RNA (snoRNA), and so on [2]. MiRNA, processing from NcRNAs, is approximately 22 nucleotide (nt) long, single-stranded and endogenous [3]. MiRNAs are processed into double-stranded complexes by Drosha and Dicer from hairpin precursors. One of the strands binds to Argonaute to form an RNA-induced silencing complex (RISC), which guides the complex to target messenger RNAs (mRNAs) to direct translational silencing or mRNA degradation. The remaining strand, called miRNA-star strand (miR*), would either be degraded or accumulates at low levels in most cases [4]. In organisms, miRNA regulates gene expression through binding usually at the partially complementary sites in 3’ untranslated region (3’ UTR) of target mRNAs, and causing inhibition of translation or mRNA degradation in post-transcriptional gene expression regulation [5].

The carmine spider mite, *Tetranychus cinnabarinus*, is an important pest which seriously does serious harm to more than one hundred agriculture crops like cotton, beans, and so on [6]. Its control is largely based on the use of chemical insecticides and acaricides. However, due to its high reproductive capacity, strong adaptability and short life cycle, *T*. *cinnabarinus* has developed resistance to these acaricides rapidly, thus making the control even more difficult [7].

*Tetranychus urticae*, the sibling species of *T*. *cinnabarinus*, is also a distributed polyphagous pest mite. Because *T*. *cinnabarinus* and *T*. *urticae* are very similar in external morphologies, biological, and molecular characteristics, they were also considered as two forms (red and green) of a single species (*T*. *urticae*) [8, 9]. However, in terms of hybridization, changes in body color, body size, external morphological features, ultrastructure, physiology and biochemistry, and ecology, they are two different species [10, 11]. *T*. *urticae*, along with the genome sequencing, got a final set of 226829 unique sRNAs that mapped to 676266 different loci in the genome and 52 sRNAs were predicted to be miRNAs in *T*. *urticae* [12]. Also in *T*. *urticae*, there were 91 and 20 miRNAs differentially expressing in response to Wolbachia infection in female and male mites, respectively, and these miRNAs negatively regulated 90 mRNAs in females and 9 mRNAs in males [13]. In *Panonychus citri*, 594 known miRNAs grouped into 206 families and 31 novel miRNAs in the four developmental stages were identified [14]. In insects, some reports proved that miRNAs were involved in the formation of insecticide resistance. For example, cpp-miR-71 might play a contributing role in the deltamethrin resistance in *Culex pipiens* [15], miR-278-3p could regulate the pyrethroid resistance in *C*. *pipiens* [16]. But there has been no report on mites miRNA associated with insecticide resistance yet.

Generally, fenpropathrin is applied as a broad-spectrum insecticide, extensively targeting in various species of mites and insects on fruit, vines, vegetables, cotton, field crops, and glasshouse crops [17–19]. There have been thorough studies on the resistance mechanism of *T*. *cinnabarinus* to fenpropathrin. A mutation in the sodium channel gene (F1538I) resulted in target resistance as well as enzyme activity increase including glutathione S-transferase (GST), cytochrome P450 (P450, CYP) and andcarboxyl/choline esterase (CCEs) are responsible for metabolic resistance [20–23]. As a key component in post-transcriptional gene expression regulation, if miRNAs are involved in fenpropathrin resistance in *T*. *cinnabarinus*?

To date, more than 30000 miRNAs have been found in over 100 organisms [24]. However, the role of miRNAs in insecticide resistance of mite has not received enough attention. In this study, we conducted sRNA libraries from the female adult of susceptible (TS) and fenpropathrin-resistant strains (TR) of *T*. *cinnabarinus*. Then we analyzed the expression profiles of the miRNAs from the two strains and predicted the targets of miRNAs by 6 algorithms, the functional annotation of the targets of differentially expressed miRNAs (DEmiRNAs) were also performed. These results will be extremely helpful to investigate the roles of miRNAs in the formation of fenpropathrin resistance of *T*. *cinnabarinus*.

## Material and Methods

### Ethics Statement

The laboratory population of carmine spider mite (CSM), *T*. *cinnabarinus* was collected from the field of Beibei District, Chongqing municipality, China. There was no specific permission required for these collection activities because this mite is a kind of agriculture-harmful pest and distributes worldwide. We confirmed that the field collection did not involve endangered or protected species.

### Mite Strains

TS strain of *T*. *cinnabarinus* was originally obtained from the field of Beibei District, Chongqing, China, which has been maintained without any acaricide treatments for 15 years. TR strain was generated from TS strain and had been subsequently selected >70 generations (resistant level more than 100-fold) by fenpropathrin to maintain resistance, the detailed resistance screening methods refer to He et al [22]. All strains feeding on fresh potted cowpea leaves were kept in artificial climate chamber under the conditions of 26 ± 1°C, 55–70% relative humidity and a 14 h light /10 h dark cycle.

### Small RNA Library Development and Sequencing

Five hundred TS and TR 3–5 days female adults were collected for total RNA extraction using Trizol (Invitrogen, USA). The integrity and purity of the total RNA were confirmed by 1% agarose gel electrophoresis and NanoVue UV-Vis spectrophotometer (GE Healthcare Bio-Science, Uppsala, Sweden). Moreover, the RNA integrity number (RIN) was measured by Novogene Company (NC). We conducted two biological repeats for each strain, the correlation analysis between the two biological repeats was detected by NC.

Qualified total RNA was used to construct the sRNA libraries with TruSeq small RNA Sample Pre Kit (Illumina). Briefly, total RNA was ligated to 5’ and 3’ adaptors, then cDNA was synthesized by reverse transcription. After PCR amplification of the cDNAs, the amplified PCR products within 130–160 bp were separated and purified by a 6% polyacrylamide gel. The Agilent Bioanalyzer 2100 system was used to assess the library quality using DNA High Sensitivity Chips. The qualified libraries were sequenced on a HiSeq2000 sequencer (Illumina).

### Bioinformatics Analysis

After Illumina sequencing, raw data were obtained after the original image data transferred into sequence data through base calling [25]. Then the raw data were processed through NC’s Perl and Python scripts. In order to obtain clean reads, we removed the low quality reads and trimed 3’joined sequence. Moreover, the reads containing more than two N (undetermined bases) and poly A or T or G or C, with contamination of adaptor sequences, without 3’adapter or the insert tag were removed from the raw data. Next, we selected sRNAs with lengths of 18–30 nt for further analysis.

In order to annotate all the sRNAs, the Bowtie [26] was used to map the sRNA tags to the *T*. *urticae* genome (http://bioinformatics.psb.ugent.be/orcae/overview/Tetur) and analyze the expression and distribution of the mapped sRNAs on the reference sequence. Then the mapped sRNAs were used to do a blastn search against the miRNA precursor of *T*. *urticae* in the miRNA database (miRBasev. 20.0; released in June, 2013) to obtain the known miRNA, only perfectly matches were accepted and retained for the next analysis. Next, the remained sRNAs were mapped to the NcRNA annotation database of *T*. *urticae* (https://bioinformatics.psb.ugent.be/gdb/tetranychus/small_RNAs/) to remove rRNAs, tRNAs, snRNAs and snoRNAs, the repeat sequences database (http://www.repeatmasker.org/cgi-bin/WEBRepeatMasker/) was used to filter tags originating from repeat sequences. Moreover, the remained sRNAs were mapped to the exon and intron of mRNAs of *T*. *urticae* to remove the tags from the degradation of protein-coding genes. At last, the remained sRNAs were used to predict the novel miRNA through the two available software miREvo [27] and mirdeep2 [28]. The criteria of the novel miRNAs: A. the ~22-nt sequence can be identified in a library of cDNAs made from size-fractionated RNA. Which must precisely match the genomic sequence of the organism from which they were cloned; B. Prediction of a potential 60–80 nt fold-back precursor structure with the lowest free energy could be partitioned into candidate mature, loop and star part based on the reads mapping to it. It should not contain large internal loops or bulges, particularly not large asymmetric bulges. In addition, the predicted fold-back precursor secondary structure of the phylogenetic conserved ~22-nt miRNA sequence could be partitioned into candidate mature, loop and star part, too, but need not be the lowest free energy folding alternative; C. Detection of increased precursor accumulation in organisms with reduced Dicer function [29]. In addition, statistics of the length and count of these miRNAs sequence in four libraries were conducted.

### Amplification of the miRNA Precursors

Genomic DNA was extracted from the female adults using DNeasy Blood & Tissue Kit (QIAGEN). According to the sequences of the precursors of the novel miRNAs, primers for 8 random precursors of novel miRNAs were designed by Primer Premier 5.0 (Premier Biosoft International, Palo Alto, CA, USA). Fragments were amplified by PCR and the products were examined by 3% agarose gels. The sequences of the primers were shown in the S1 Table.

### Expression Profile Analysis

In order to analyze the expression profiles of the miRNAs of two strains, the read counts of miRNAs were normalized into TPM (transcript per million) through the Normalization formula: Normalized expression = (mapped read count/Total reads) × 1000000 [30]. Then we used the package DESeq to analyze the DEmiRNAs between libraries [31], the P value was all adjusted by Benjamini–Hochberg false discovery rate (FDR) procedure [32], the threshold for significant differential expression by default was P < 0.05 and |log_2_ (fold change)| > 1, the log_2_-ratio plot was then generated. In addition, the TPM of significantly DEmiRNAs in two strains were used for hierarchical cluster analysis [33].

### Target Prediction

Because the genome of *T*. *cinnabarinus* was not available, the 3’UTR annotation information originated from the genome database of *T*. *urticae* was used to predict target genes of miRNA through miRanda [34], findtar [35], microtar [36], PITA [37], RNA22 [38] and RNAhybrid [39]. In addition, for target gene functional annotation, the target genes of DEmiRNAs were used for gene ontology (GO) enrichment analysis [40].

### Stem-Loop Quantitative RT-PCR Assay

The expression levels of miRNAs in the two strains were verified by stem-loop RT-PCR [41]. Briefly, total RNA from two strains was extracted by TRIZOL, respectively, then the DNAse I (Promega, Madison, WI, USA) was used to treat the total RNA. Reverse transcription was then performed using PrimeScript 1st Strand cDNA Synthesis Kit (Takara Biotechnology Dalian Co., Ltd., Dalian, China) following the manufacturer's instructions with looped antisense primer. A MX3000P thermal cycler (Agilent Technologies, Inc., Wilmington, NC, USA) was used to perform the RT-qPCR with iQ^™^SYBR Green Supermix (BIORAD, Hercules, CA, USA), the optimized amplification protocol was: 94°C for 2 min, followed by 40 cycles by 94°C for 10 s, 60°C for 20 s and elongation at 72°C for 10 s, melt curve analyses (from 60 to 95°C) were included in the end to ensure the uniqueness of the amplified products. RPS18 (FJ608659) was used as stable reference gene [42, 43]. The 2^−ΔΔCt^ method was used to analyze the quantification of expression level [44]. There were three biological repeats with three technical replicates in each experiment. All the primers are shown in S2 Table.

## Results

### Sequencing of sRNAs in *T*. *cinnabarinus*

**Fig 1:**
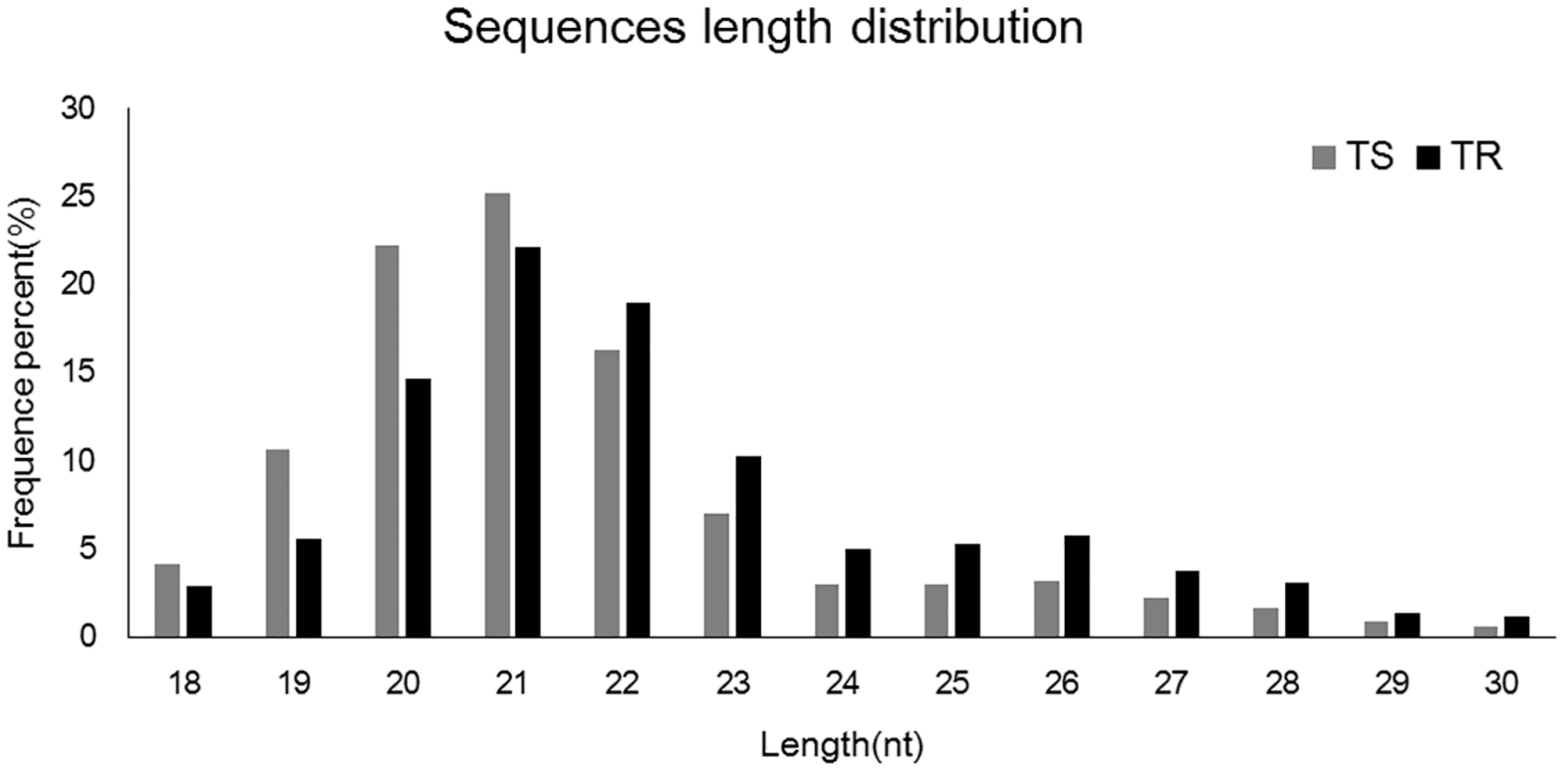
Small RNA (sRNA) length distribution in TS and TR libraries. TS: susceptible strain, TR: fenpropathrin-resistant strain.

All the parameters proved that the quality of the total RNA met the requirement of the sRNA deep sequencing (S3 Table), and the repetitiveness of the two libraries of each strain had excellent repeatability (S1 Fig). The raw data of the four libraries had been deposited in NCBI’s Sequence Read Archive (SRA) under accession number SRP067789. We obtained 11883903 and 12786631 total reads from TS and TR libraries, respectively. 11719385 and 12474785 clean reads were filtered out from TS and TR libraries through the sequencing process (Table 1). The length of the sRNAs from TS and TR libraries ranged from 18 to 30 nt, and the peak size was 21 nt, followed by 20 and 22 nt (Fig 1).

**Table 1:** Output statistics of the *T*. *cinnabarinus* small RNA sequencing.

Sequencing	TS (Count)	Percent (%)	TR (Count)	Percent (%)
Total Raw Reads	11883903	100%	12786631	100%
N>10%	11674	0.10%	13588	0.11%
Low quality	3984	0.03%	5417	0.04%
5’adapter	5163	0.04%	10035	0.08%
3’adapter	117068	0.99%	241376	1.89%
Ploy A/G/C/T	26629	0.22%	41430	0.32%
Total Clean Reads	11719385	98.62%	12474785	97.56%

**Fig 2:**
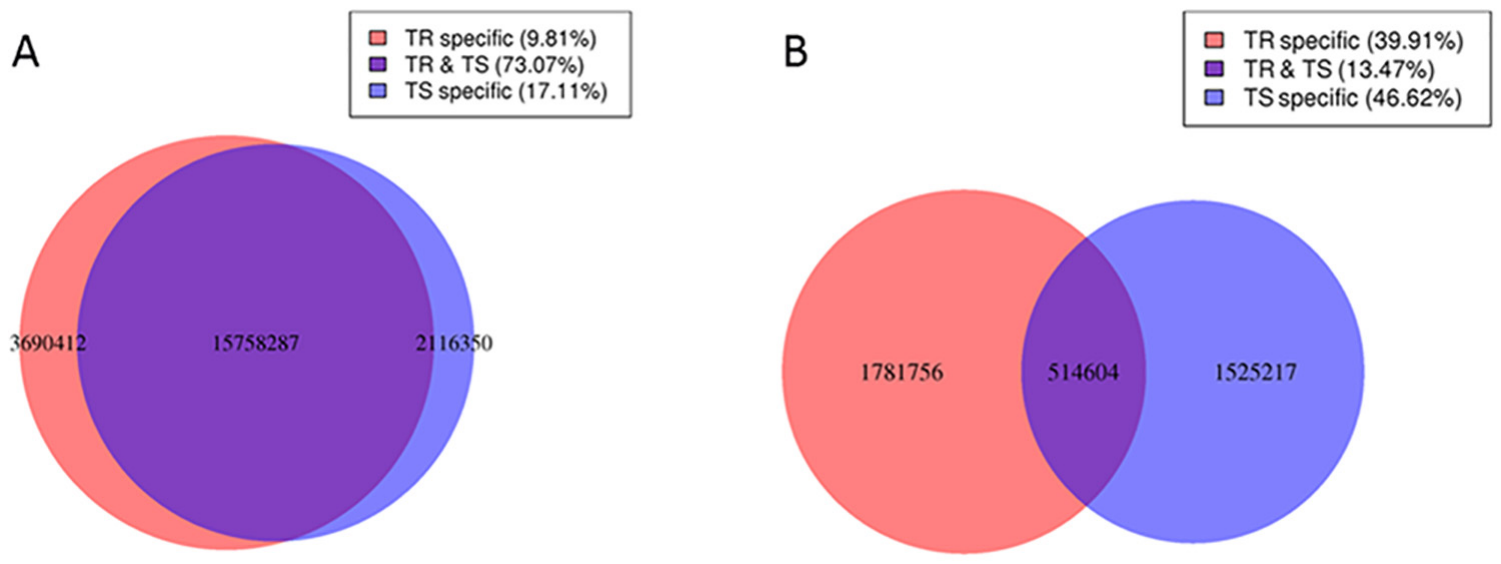
Analysis of the specific and common sequences in TS and TR libraries. (A) Analysis of the amount of the specific and common sequences between two libraries. (B) Analysis of the species of the specific and common sequences between two libraries.

Furthermore, we counted the amount and species of the common and specific sRNAs between TS and TR libraries. The amount of the common sRNAs sequences between TS and TR libraries accounted for 73.07% in total sRNAs from the two libraries, while the amount of specific sRNAs sequences in TS and TR library accounted for 17.11% and 9.81%, respectively. The species of the common sRNAs between TS and TR libraries accounted for 13.47% in total sRNAs from the two libraries, however, the species of specific sRNAs in TS and TR libraries accounted for 46.62% and 39.91%, respectively (Fig 2).

### Genome Mapping and sRNAs Annotation

**Fig 3:**
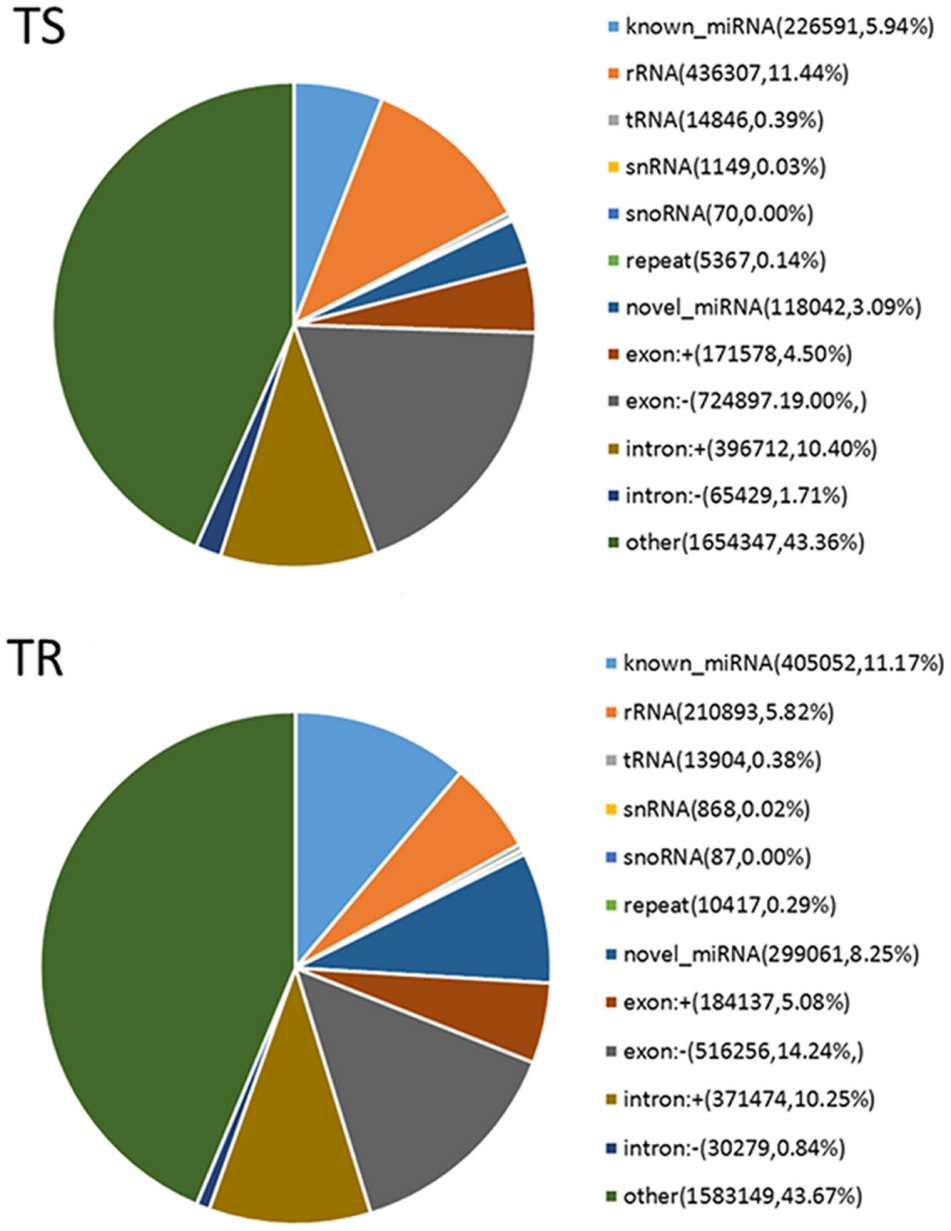
Classification of sRNAs in the TS and TR libraries. Other, unannotated sRNA.

After mapping the total clean reads to the *T*. *urticae* genome, 3815335 genome-matched reads (36.34%) were filtered out in TS library, 3625577 genome-matched reads (32.76%) in TR library (Table 2). Then we annotated the sRNAs in the foundation of the following priority rule: known miRNA > rRNA > tRNA > snRNA > snoRNA > repeat > gene > novel miRNA. The reads of the known miRNA were 226591 (5.94%) and 405052 (11.17%) in TS and TR libraries, respectively, the reads of the novel miRNA were 118042 (5.94%) and 299061 (8.25%) in TS and TR libraries, respectively (Fig 3). NcRNA (rRNA, tRNA, snRNA and sonRNA) accounted for 11.86% and 6.22% of total mapped sRNAs in TS and TR libraries, respectively, but a large fraction was the unannotated small RNA.

**Table 2:** Genome mapping information of TS and TR libraries.

Sample	Total sRNA	Mapped sRNA	"+" Mapped sRNA	"- " Mapped sRNA
TS	10498373 (100.00%)	3815335 (36.34%)	3191921 (30.40%)	623414 (5.94%)
TR	11066676 (100.00%)	3625577 (32.76%)	2619307 (23.67%)	1006270 (9.09%)

+ indicates plus strand of the chromosome, − indicates minus strand of the chromosome.

### Known and Novel miRNAs in *T*. *cinnabarinus*

Through the bioinformatics analysis, we obtained 75 known miRNAs in the two strains. 90.67% of the known miRNAs ranged from 21 to 23 nt, the known miRNAs could be aligned to 28 miRNA families (S4 Table).

**Fig 4:**
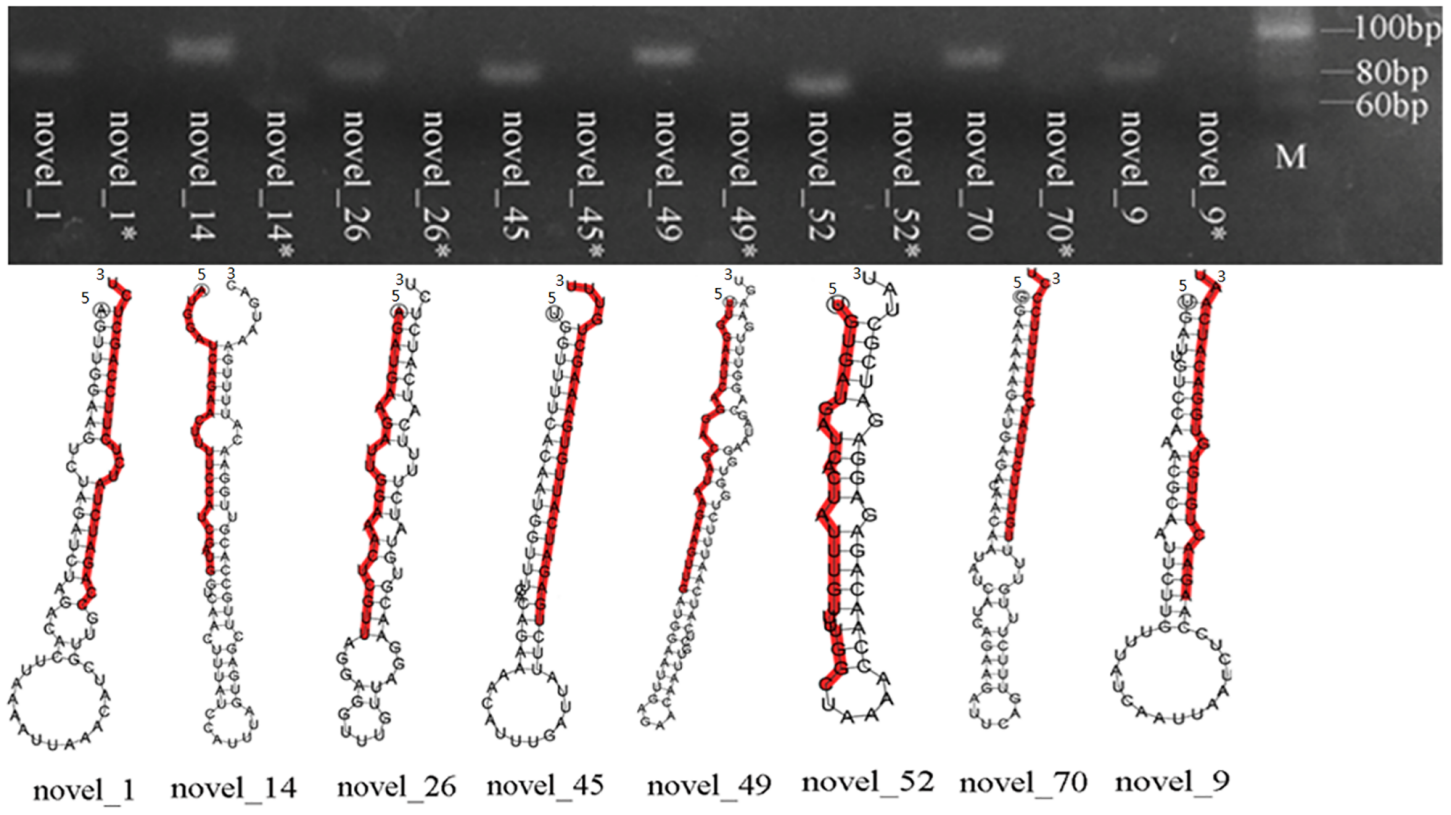
PCR analysis and predicted secondary structures of eight novel miRNAs in *T*. *cinnabarinus*. Agarose gel electrophoresis of 8 novel miRNAs. M: 20bp DNA Ladder (Dye PLus). novel_n: PCR product of the 8 miRNA precursors, novel_n*: PCR control, control reaction system: forward primer, reverse primer and enzyme.

A total of 64 novel miRNAs were found in the two strains, the length of the novel miRNAs ranged from 18 to 25 nt (S5 Table). The novel miRNAs were originated from 64 miRNA precursors, the minimum free energy of precursor secondary structure of novel miRNAs ranged from -10.6 to -35 kcal/mol, 36 novel miRNAs were located at 3’ end of the miRNA precursors. In addition, we designed 8 pairs of primers for precursors of novel miRNA. As expected, we amplified products shorter than 100 bp from the *T*. *cinnabarinus* genome (Fig 4).

Many reports reported that the nucleotide bias at each position and first nucleotide bias of miRNAs had certain rule [45]. In our study, the uracil (U) was the most used of the first 22 nt of the novel miRNAs. On the contrary, the cytosine (C) was the least used base. The uracil (U) was the most used in the first base of the novel miRNAs, the adenine (A) was the most used in the first base of the 24 nt novel miRNAs, the guanine (G) was the least appeared in the first base of the novel miRNAs (S2 Fig).

### Target Gene Prediction

The target gene prediction results for all the miRNAs were shown in S6 Table. MiRanda, findtar, microtar, PITA, RNA22 and RNAhybrid were used to predict the target gene of the known and novel miRNAs, which resulted in 26196, 19310, 11182, 19455, 4951 and 17249 miRNA-target pairs, respectively. There were 10649 miRNA-target pairs collectively predicted by 2 algorithms, 2026 miRNA-target pairs for 3 algorithms, 344 miRNA-target pairs for 4 algorithms, 37 miRNA-target pairs for 5 algorithms, and tci-miR-210-3p and tetur10g02710 was collectively predicted for 6 algorithms (S7 Table). In total, 78592 miRNA-target pairs were predicted through 6 algorithms.

### Differential Expression Analysis and RT-qPCR Validation

**Fig 5:**
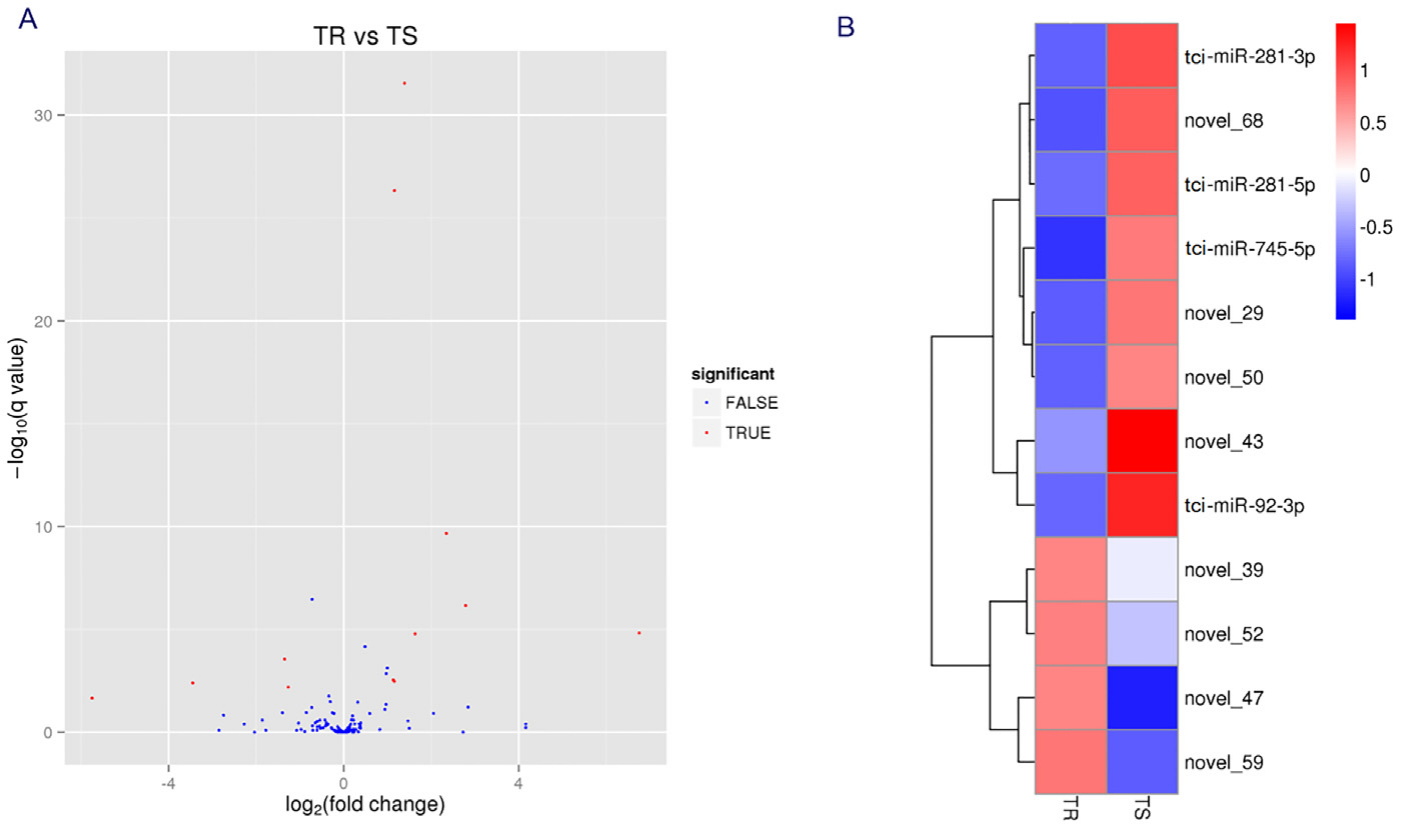
DEmiRNAs between TS and TR strains. (A) Comparison of the expression levels of known and novel miRNAs between TS and TR strains. Each point represents a miRNA. The x-axis shows the expression difference (log_2_ (fold change)) in two strains, the y-axis shows the statistically significant degree (-log_10_ (q value)) of expression difference. Red points represent significantly DEmiRNAs between two strains, blue points represent the miRNAs with similar expressing level between two strains. (B) Cluster analysis of different expressed miRNAs, the cluster analysis is based on the log_10_ (TPM+1) of the DEmiRNAs in two strains. Red represent the high expression miRNAs, blue represent the low expression miRNAs.

The expression of 12 miRNAs were significantly different when comparing the expression profiles between TS and TR libraries (Fig 5), the detailed expression level of the known and novel miRNAs in TS and TR libraries were shown in S8 Table, and the results have been deposited in NCBI’s Gene Expression Omnibus (GEO) under accession number GSE76584. Novel_39, novel_47, novel_52 and novel_59 were significantly up-regulated in TR library, tci-miR-281-5p, tci-miR-281-3p, tci-miR-745-5p, tci-miR-92-3p, novel_29, novel_43, novel_50 and novel_68 were significantly down-regulated in TR library.

**Fig 6:**
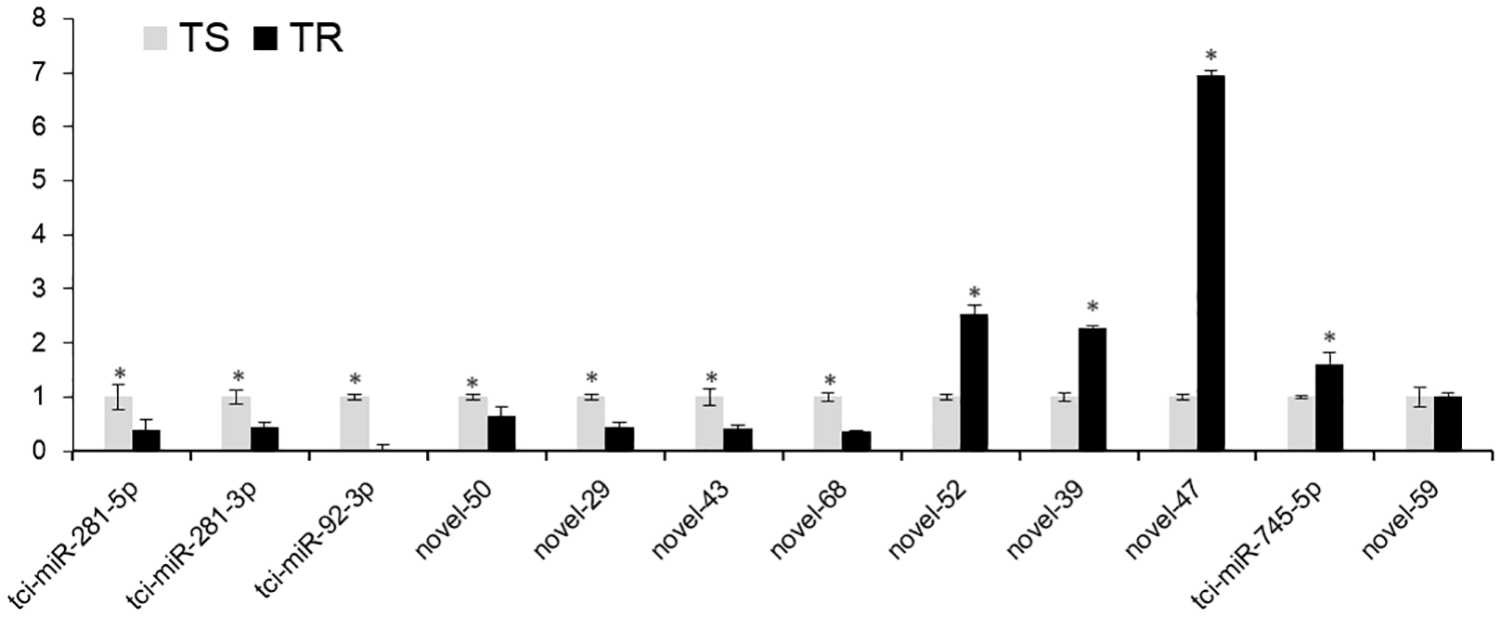
Expression patterns of DEmiRNAs in TS and TR strains. The x-axis indicates miRNAs and the y-axis indicates the relative expression level in TR strain. For each miRNA, the expression level in TS strain was set as 1.

Moreover, we used the stem-loop RT-PCR to measure the expression levels of the 12 significantly DEmiRNAs, tci-miR-281-5p, tci-miR-281-3p, tci-miR-92-3p, novel_50, novel_29, novel_43 and novel_68 were significantly down-regulated in TR strain, novel_39, novel_52, novel_47 and tci-miR-745-5p were significantly up-regulated in TR strain, but novel_59 was no significantly differences between two strains. The accordant rates of Illumina sequencing and RT-qPCR reached 83.3% (Fig 6).

### Functional Analysis of Predicted Targets for DEmiRNAs

**Fig 7:**
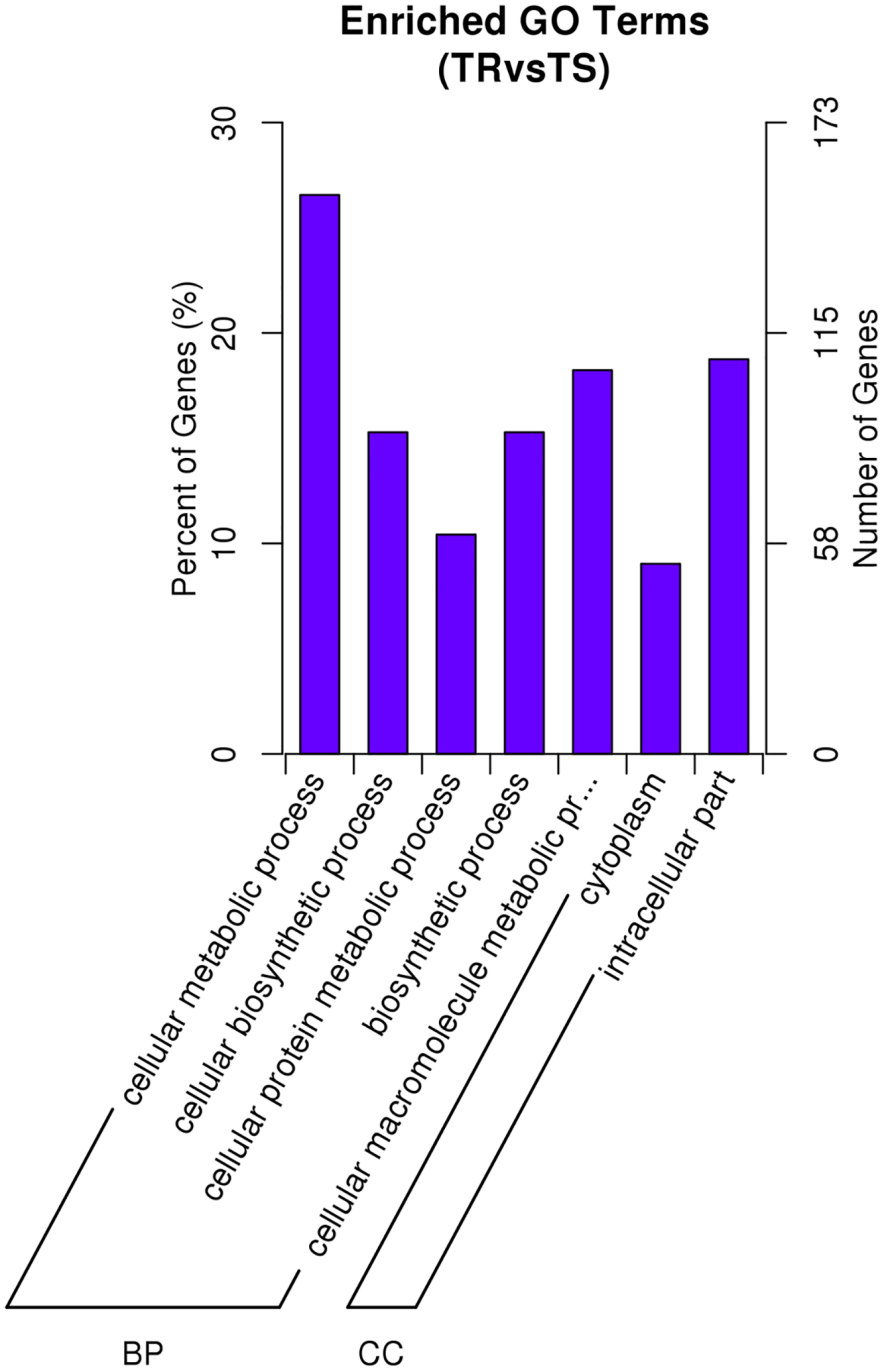
GO analysis results of the target genes of DEmiRNAs. The x-axis is the GO category and the y-axis is the percent and number of genes. BP: Biological Process, CC: Cellular Component.

Furthermore, we analyzed the biological functions of the target genes of DEmiRNAs, the GO annotation enrichment results showed that most of these genes related to metabolic and biosynthetic processes (Fig 7). In addition, partial of predicted target genes of DEmiRNAs belong to three detoxification enzyme families (P450, GST, CCE). These genes were listed in Table 3, the predicted targets of 8 down-regulated miRNAs in TR libraries included 13 CCE genes, 5 GST genes and 4 P450 genes, the predicted targets of 4 up-regulated miRNAs contained 8 CCE genes, 3 GST genes and 5 P450 genes.

**Table 3:** The potential insecticide-related targets of the DEmiRNAs.

MiRNA	Log_2_ (TR/TS)	Target (Detoxification enzymes)
tci-miR-281-5p	-1.39	TuCCE-29, TuCCE-42, TuCCE-52, TuCCE-02
tci-miR-281-3p	-1.16	TuGSTm01, TuGSTm04, CYP392E9, TuCCE-61, TuCCE-63, CCEinc-09, TuCCE-48
tci-miR-92-3p	-2.35	TuGSTd01, TuCCE-29, TuCCE-02, CCEinc-09
novel_29	-6.75	TuGSTd01, CYP392A10, CYP392D1, TuCCE-29, TuCCE-40, TuCCE-45, TuCCE-38
novel_43	-2.79	CYP307A1, TuCCE-29, TuCCE-02
novel_50	-1.13	CYP307A1
novel_68	-1.63	TuGSTz01, TuGSTm06, TuCCE-29, TuCCE-15, TuCCE-63
tci-miR-745-5p	-1.16	TuGSTd01, CYP392D1, TuCCE-58
novel_39	1.35	TuGSTd16, CYP307A1, CYP392E7, TuCCE-22, TuCCE-63
novel_47	1.27	CYP392A10, CYP392E7, TuCCE-28, TuCCE-21, TuCCE-29
novel_52	3.45	TuGSTm04, TuGSTm05, CYP307A1, CYP391A1, CYP315A1, TuCCE-29, TuCCE-02, TuCCE-45, TuCCE-16
novel_59	5.75	TuCCE-29

## Discussion

MiRNAs comprise a large family of endogenous and evolutionarily conserved NcRNAs that post-transcriptionally regulate mRNAs and influence fundamental cellular processes and gene expression programs in metazoan animals, plants and protozoa. Over the years many publications have reported the miRNAs in insect and their role in diverse functions, such as development and host-microorganism interactions [46–49]. As more and more reports about miRNAs were reported, the miRNAs in mites have also made certain progress. In our study, we used Illumina sequencing to identify the miRNAs in *T*. *cinnabarinus*, 75 known miRNAs were detected in the *T*. *cinnabarinus*, which could be aligned to 28 miRNA families, while in *T*. *urticae*, the miRNAs of the four developing stages were classified into 43 miRNA families [12]. The known miRNAs in other developing stages still need further exploration, the differential distribution of miRNAs in different developing stages of insect have got a evidence in *Bombyx mori*, only 106 miRNAs expressed in all stages of development, but the number of egg- and pupa-specific miRNAs was up to 248 [50]. In addition, 64 novel miRNAs were identified in *T*. *cinnabarinus*, the characteristic signatures of the novel miRNAs met the criteria described previously [29]. The first base of the novel miRNAs of *T*. *cinnabarinus* was mainly the uracil (U), which was in accordance with the first base preference of miRNAs in other reports [45], these novel miRNAs were complementary to the miRNA in mites.

With the deepening of the study of miRNA function, the study of miRNAs in cancer has clearly established that miRNA-mediated alterations in levels of drug targets, drug transporters, metabolic enzymes or cell apoptosis proteins can lead to drug resistance [51]. Altered gene expression associated with drug resistance in other systems indicated that examination of miRNA activity and 3′-UTR interactions in parasitic nematodes was warranted to improve the understanding of drug resistance mechanisms [52]. Some reports proved that miRNAs involved in insecticide resistance, for example, in *Cx*. *pipiens*, 28 differentially expressed miRNAs between deltamethrin-sensitive and resistant strains were expected to contribute to the development of pyrethroid resistance [15]. In our study, the reads of known and novel miRNAs in TR library were two fold of the reads of miRNAs in TS library, which indicated that the different expression of miRNAs might contribute to the formation of the fenpropathrin resistance.

After normalization, we found 12 miRNAs that were significantly differently expressed between TS and TR strains. tci-miR-281-5p and tci-miR-281-3p were belonged to mir-46 family, which was reported to function in juvenile hormone and/or ecdysone mediated signaling pathways and to modulate the development of wings, legs and neuronal system in insect [53]. MiR-281 also might participate in the expression regulation of immunity-related genes in *Manduca sexta* [54], so we speculate that tci-miR-281 might play a role in the development and immunity process in *T*. *cinnabarinus*. tci-miR-92-3p was belong to mir-25 family, most reports about the function of mir-25 focused on mammalian cell, for example, mir-25 regulated pigmentation in alpaca skin melanocytes [55], reduced cardiac function during heat failure [56] and increased cell proliferation by negative regulation of an isoform of the cell-cycle regulator p63 [57]. In addition, there were a few reports about the function of miR-92 in insect. The targets of miR-92 may participate in mediating flavivirus infection of mosquito host [58], and in *B*. *mori*, miR-92 is associated with embryogenesis, a stage of high cellular proliferation and differentiation. But how these significantly DEmiRNAs specifically involving in the formation of the fenpropathrin resistance requirs a further study.

MiRNAs achieve their functions through working as the post-transcriptional regulator of the target genes. So to identify the targets of these DEmiRNAs was an important foundation for clarifying the relationship between these miRNAs and insecticides resistance. Study on insecticides resistance had demonstrated that activation of detoxification enzymes and mutation of sodium channels could contribute to the development of pyrethroid resistance [59]. MiRNA negative regulates the expression of their targets by causing inhibition of translation or mRNA degradation, so we analyzed the detoxification enzyme target genes of the significantly down-regulated miRNAs in TR strain. CYPs in families 1–4 are critical and often inducible components of phase I detoxification systems of vertebrates, invertebrates and plants [60–63]. Among these predicted P450s targeted by DEmiRNAs, the 5 P450 genes belong to CYP2 clan, which was responsible for essential physiological functions, e.g. ecdysone metabolism and juvenile hormone biosynthesis. In *T*. *urticae*, CYP392E7 and CYP392E9 had been reported might participate in the formation of the avermectin resistance and CYP392E9 also might be related with the spirotetramat resistance [64]. Elevated GSTs in the resistant strains attenuated the pyrethroid-induced lipid peroxidation and reduced mortality [65]. In our study, most of the predicted target GST genes were belong to class mu and delta, which seem to be implicated in xenobiotic detoxification in mite [66]., A predicted DEmiRNAs targeting gene, GSTd01, had been reported to be related to the fenpropathrin resistance in previous study of *T*. *cinnabarinus* [67]. For most pyrethroids, CCEs hydrolysis also is important for detoxification than the oxidation [68]. In mites, the CCE genes were reported to participate in the insecticide resistance and a previous study had detected overexpression of TCE2 (homology with TuCCE-55) in the fenpropathrin resistant *T*. *cinnabarinus* [20]. But none of the 12 DEmiRNAs in TR strain targeted on TCE2 gene, which suggests the overexpression of TCE2 gene may be regulated by other mechanism. And some detoxification enzyme target genes might be predicted by the up-regulated miRNAs in TR strain too, which may be play a role in maintaining a balance to a certain extent during the cell growth and development. Although the miRNAs of *T*. *cinnabarinus* might function in the formation of the fenpropathrin resistance through regulating the insecticide resistance related genes, the authenticity of these miRNA-target pairs still need further experimental validation.

Besides miRNA, several classes of small non-coding RNA were identified in our study. For example, the reads of rRNA in TS library were higher than the reads of rRNA in TR library, further investigation of these sRNAs could be helpful in improving our understanding of mechanisms of insecticide resistance.

## Conclusion

We constructed sRNA libraries from the female adult of TS and TR strains and identified 75 known miRNAs and 64 novel miRNAs, of which, 12 miRNAs were significantly differentially expressed between TS and TR strains. The target genes of known and novel miRNAs were predicted using 6 algorithms. GO annotation for the targeted genes of DEmiRNAs was performed and the results showed that most of the target genes related to metabolic and biosynthetic processes. In addition, functional study of the differently expressed miRNAs targeting genes belonging to detoxifying enzyme families (P450, GST and CCE) indicated these miRNAs targeting genes might involve in fenpropathrin resistance. These results provide important clues for further study on the mechanisms of miRNA involved in fenpropathrin resistance and for putting forward new strategy on resistance management in *T*. *cinnabarinus*.

## Supporting Information

S1 Fig Correlation analysis of miRNA expression between the samples. (TIF)

S2 Fig Analysis of nucleotide bias of the novel miRNA candidates of TS and TR libraries in *T*. *cinnabarinus*. (TIF)

S1 Table Primers used for eight novel miRNA precursors. (DOCX)

S2 Table Primers used for reverse transcription and RT-qPCR. (DOCX)

S3 Table Total RNA quality indicator of *T*. *cinnabarinus*. (DOCX)

S4 Table The known miRNAs in TS and TR strains. (DOCX)

S5 Table The novel miRNAs and their precursors in TS and TR strains. (XLSX)

S6 Table Target gene prediction for miRNAs with six algorithms. (XLSX)

S7 Table The miRNA-target pairs collectively predicted by more than 2 algorithms. (XLSX)

S8 Table The expression level comparison results of the known and novel miRNAs in TS and TR strains. (DOCX)
