# Supplementary figures and images for: Identification of Differentially Expressed microRNAs between the Fenpropathrin Resistant and Susceptible Strains in Tetranychus cinnabarinus
Source: PLoS One. 2016 Apr 6;11(4):e0152924. doi: 10.1371/journal.pone.0152924 (PMC4822788; doi:10.1371/journal.pone.0152924)

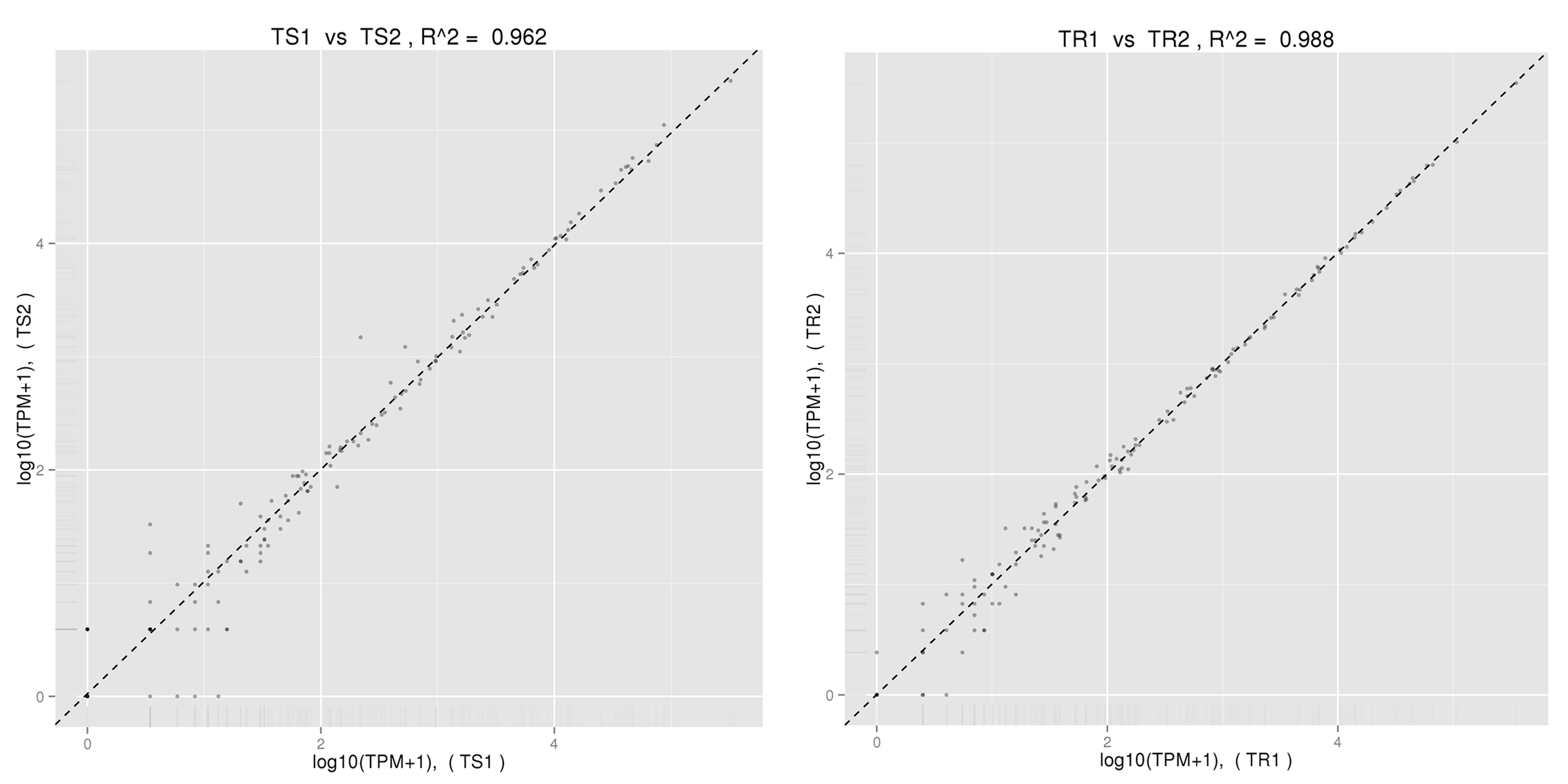

Supplement: S1 Fig — (TIF) [file pone.0152924.s001.tif]

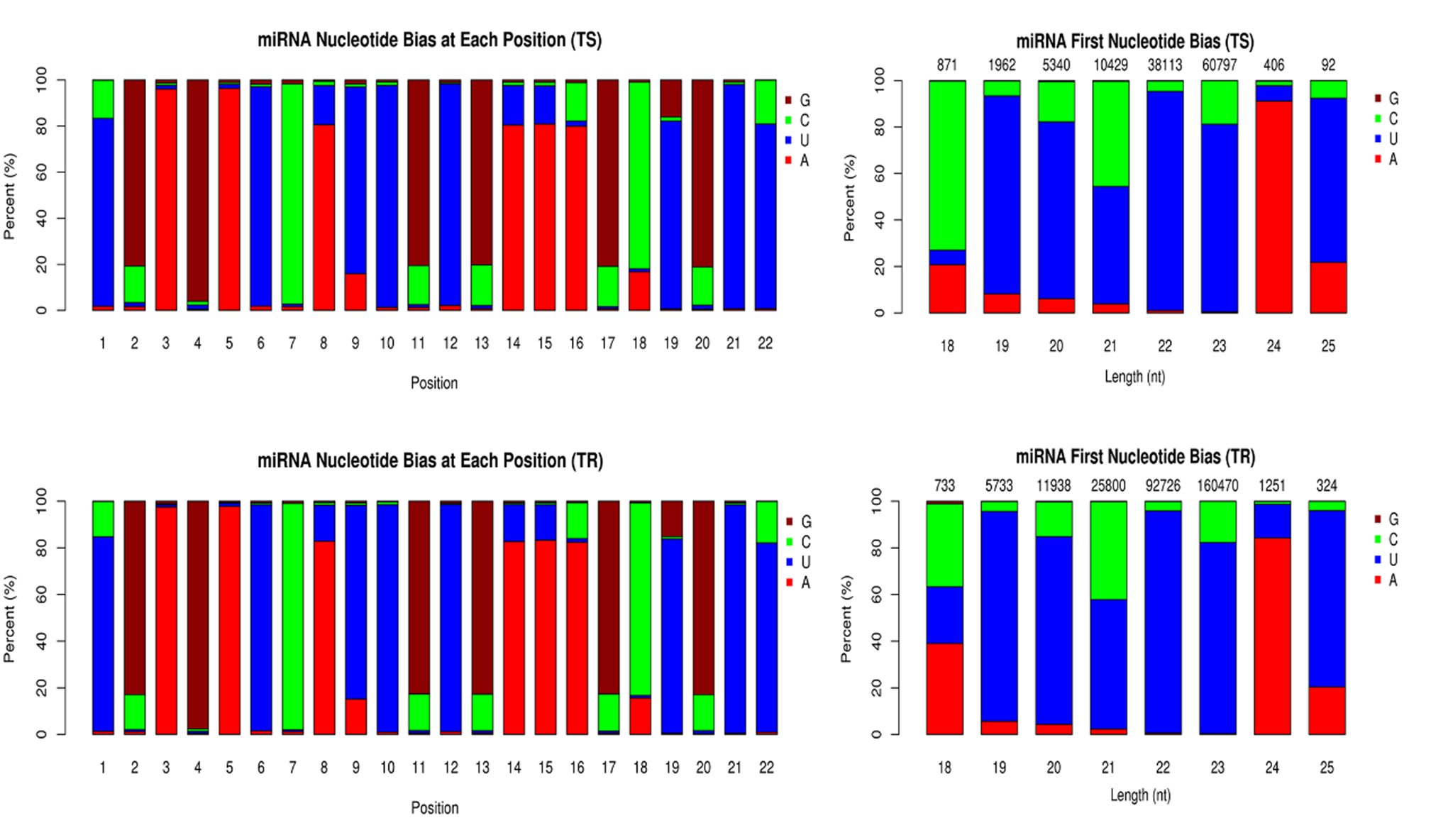

Supplement: S2 Fig — (TIF) [file pone.0152924.s002.tif]
