# Supplementary material for: Identification of Differentially Expressed microRNAs between the Fenpropathrin Resistant and Susceptible Strains in Tetranychus cinnabarinus
Source: PLoS One. 2016 Apr 6;11(4):e0152924. doi: 10.1371/journal.pone.0152924 (PMC4822788; doi:10.1371/journal.pone.0152924)
Supplement: S1 Table — (DOCX) [file pone.0152924.s003.docx]

**S1 Table. Primers used for eight novel miRNA precursors**

| **Primer name** | **Sequence (5'-3')** |
| --- | --- |
| novel_1-F | AGTTGGAAGTCTAGATCTAG |
| novel_1-R | AGAGCTGGAAGAGATAGAT |
| novel_14-F | ATGGATCAGAACTTTTCCAT |
| novel_14-R | GTCATTTCAAAATGTTCCA |
| novel_26-F | AGATGAAGATTGGAAACTC |
| novel_26-R | AGAGATGATGAAAAGATACA |
| novel_45-F | TGGTTTTCACAATGGTTT |
| novel_45-R | AAACAGCTTTCACAATGAT |
| novel_49-F | TTGGAATCAGGACGATAA |
| novel_49-R | ACTTCAAACCTGCTATTCC |
| novel_52-F | GCTGTGATGATCACTATTTG |
| novel_52-R | ATAGCGATCTCCTCTCTGTT |
| novel_70-F | GGAAAAAGATGAGACAAC |
| novel_70-R | AGGGAAAAAGGATAAGA |
| novel_9-F | TGATTGTCCAAACGCAA |
| novel_9-R | ATTGATGTCCACACACAGT |
