# Supplementary material for: Identification of Differentially Expressed microRNAs between the Fenpropathrin Resistant and Susceptible Strains in Tetranychus cinnabarinus
Source: PLoS One. 2016 Apr 6;11(4):e0152924. doi: 10.1371/journal.pone.0152924 (PMC4822788; doi:10.1371/journal.pone.0152924)
Supplement: S2 Table — (DOCX) [file pone.0152924.s004.docx]

**S2 Table.** **Primers used for reverse transcription and RT-qPCR**

| **Primer Name** | **Primer Sequences (5' to 3')** |
| --- | --- |
| tci-miR-281-5p-RT | CTCAACTGGTGTCGTGGAGTCGGCAATTCAGTTGAG CATCTGTC |
| tci-miR-281-5p-FP | ACACTCCAGCTGGGAAGAGAGCTTATCCGT |
| tci-miR-281-3p-RT | CTCAACTGGTGTCGTGGAGTCGGCAATTCAGTTGAG AGAGAAAG |
| tci-miR-281-3p-FP | ACACTCCAGCTGGGTGTCATGGAGTTGCTC |
| tci-miR-92-3p-RT | CTCAACTGGTGTCGTGGAGTCGGCAATTCAGTTGAGGCCGGACA |
| tci-miR-92-3p-FP | ACACTCCAGCTGGGTATTGCACTCGTCCCG |
| tci-miR-745-5p-RT | CTCAACTGGTGTCGTGGAGTCGGCAATTCAGTTGAGACGTTCCG |
| tci-miR-745-5p-FP | ACACTCCAGCTGGGAGTCCGTCTCTGGGCTG |
| novel_43-RT | CTCAACTGGTGTCGTGGAGTCGGCAATTCAGTTGAGTTGGAGGT |
| novel_43-FP | ACACTCCAGCTGGGCTCTCGGTGG AA |
| novel_29-RT | CTCAACTGGTGTCGTGGAGTCGGCAATTCAGTTGAGTCCCGACA |
| novel_29-FP | ACACTCCAGCTGGGTGACAGGGTCCT AG |
| novel_68-RT | CTCAACTGGTGTCGTGGAGTCGGCAATTCAGTTGAGCGTGGAGA |
| novel_68-FP | ACACTCCAGCTGGGCACCGGGATGATG GC |
| novel_50-RT | CTCAACTGGTGTCGTGGAGTCGGCAATTCAGTTGAGAATGCTAA |
| novel_50--FP | ACACTCCAGCTGGGTTTTCGGATTATCC TT |
| novel_39-RT | CTCAACTGGTGTCGTGGAGTCGGCAATTCAGTTGAGGCCTGCAA |
| novel_39-FP | ACACTCCAGCTGGGTTAGAAAAGAACTC CG |
| novel_52-RT | CTCAACTGGTGTCGTGGAGTCGGCAATTCAGTTGAGCAAAACCG |
| novel_52-FP | ACACTCCAGCTGGGTGTGATGATCACTATTT GT |
| novel_47-RT | CTCAACTGGTGTCGTGGAGTCGGCAATTCAGTTGAGACTTCTGA |
| novel_47-FP | ACACTCCAGCTGGGTAGTTCTGTTGGTGAT TG |
| novel_59-RT | CTCAACTGGTGTCGTGGAGTCGGCAATTCAGTTGAGGGTTAGCA |
| novel_59-FP | ACACTCCAGCTGGGTCATTACTGTCAAGAAA CC |
| RPS18-R | TGCCTATTCAAGAACCAAAGTGGG |
| RPS18-F | ACGTGCTGGTGAACTTACCGAAGA |
| Universal Primer | TGGTGTCGTGGAGTCG |

-RT means that primer is used in reverse transcription reaction for each miRNAs, -FP indicates forward primer.
