# Supplementary material for: Identification of Differentially Expressed microRNAs between the Fenpropathrin Resistant and Susceptible Strains in Tetranychus cinnabarinus
Source: PLoS One. 2016 Apr 6;11(4):e0152924. doi: 10.1371/journal.pone.0152924 (PMC4822788; doi:10.1371/journal.pone.0152924)
Supplement: S3 Table — (DOCX) [file pone.0152924.s005.docx]

**S3 Table. Total RNA quality indicator of *T. cinnabarinus***

| **Quality index** | **TS1** | **TS2** | **TR1** | **TR2** |
| --- | --- | --- | --- | --- |
| Concentration(ng/uL) | 1680 | 1610 | 1660 | 1880 |
| Volume(μL) | 12 | 12 | 12 | 12 |
| Total amount(μg) | 20.16 | 19.32 | 19.92 | 22.56 |
| OD260/280 | 1.92 | 1.95 | 1.98 | 1.97 |
| RIN | 6.1 | 6.1 | 6.3 | 6.3 |
