# Supplementary material for: Identification of Differentially Expressed microRNAs between the Fenpropathrin Resistant and Susceptible Strains in Tetranychus cinnabarinus
Source: PLoS One. 2016 Apr 6;11(4):e0152924. doi: 10.1371/journal.pone.0152924 (PMC4822788; doi:10.1371/journal.pone.0152924)
Supplement: S4 Table — (DOCX) [file pone.0152924.s006.docx]

**S4 Table. The known miRNAs in TS and TR strains**

| **Known miRNA** | **Sequence** | **Length** | **MiRNA family** |
| --- | --- | --- | --- |
| tci-miR-10-3p | GAAUUCGGCUCUAGAGAGGUUUG | 23 | mir-10 |
| tci-miR-10-5p | CACCCUGUAGAUCCGAAUUUGU | 22 | mir-10 |
| tci-miR-124-1-5p | CGUGUUCACUGUGUAUGUCUUG | 22 | mir-124 |
| tci-miR-124-2-5p | GUGUUCACUGUUUGCCUUCAUG | 22 | mir-124 |
| tci-miR-124-3p | UAAGGCACGCGGUGAAUGCCA | 21 | mir-124 |
| tci-miR-12a-3p | GUACUUAAUGUGAGACUCAUC | 21 | mir-12 |
| tci-miR-12a-5p | UGAGUAUUACAUCAGGUACU | 20 | mir-12 |
| tci-miR-12b-3p | UAGUACCUGGAAAAUUAACUCAC | 23 |  |
| tci-miR-12b-5p | UGAGUAUUUCUCCAGGUACUGA | 22 |  |
| tci-miR-133-3p | UUGGUCCCCUUCAACCAGCUGU | 22 | mir-133 |
| tci-miR-133-5p | AGUUGGUUGAAUCCGGGCCAAAU | 23 | mir-133 |
| tci-miR-137-3p | UUAUUGCUUGAGAAUACACG | 20 | mir-137 |
| tci-miR-137-5p | GUGUAUUCCUGGGCUAAUAAC | 21 | mir-137 |
| tci-miR-1-3p | UGGAAUGUAAAGAAGUAUGGAG | 22 | mir-1 |
| tci-miR-1-5p | CAUACUUCUGGACAUACCAUA | 21 | mir-1 |
| tci-miR-184-3p | UGGACGGAGAACUGAUAAGGGC | 22 | mir-184 |
| tci-miR-184-5p | CCUUAUCAUUUCCGCUGUCCAGA | 23 | mir-184 |
| tci-miR-190-3p | ACCAAGAAUCAGACAUAUGCUCA | 23 | mir-190 |
| tci-miR-190-5p | AGAUAUGUUUGAUAUUCUUGGUU | 23 | mir-190 |
| tci-miR-210-3p | UUGUGCGUGUUUCAAGCGGCUG | 22 | mir-210 |
| tci-miR-210-5p | AGUUGUUUGACCACGACACAAGG | 23 | mir-210 |
| tci-miR-2-3p | UAUCACAGCCAGCUUUGAUGAU | 22 | mir-2 |
| tci-miR-252-3p | UCCUGCAGCCUUGGUGCUUACU | 22 | mir-252 |
| tci-miR-252-5p | CUAAGUACUAGCGCCGCAGGAG | 22 | mir-252 |
| tci-miR-2-5p | UCGUCAAAGGGGUUGUGAAAUG | 22 | mir-2 |
| tci-miR-263a-3p | UGGUUUCUCUAGUGCUGUGA | 20 | mir-263 |
| tci-miR-263a-5p | UAUGGCACUGGAAGAAUUCACG | 22 | mir-263 |
| tci-miR-263b-3p | GUGGGUUCUUGGGUGCCAAAGA | 22 | mir-263 |
| tci-miR-263b-5p | CUUGGCACUGGAAGAAUUCACCG | 23 | mir-263 |
| tci-miR-276-3p | UAGGAACUUCAUACCAUGCUCG | 22 | mir-276 |
| tci-miR-276-5p | AGCUCGGUGUGGAGUUUCUUAC | 22 | mir-276 |
| tci-miR-278-3p | UCGGUGGGAUUUUCGUCCGUC | 21 |  |
| tci-miR-278-5p | ACGGAUAAAUAUUCCACCGAUA | 22 |  |
| tci-miR-279-3p | UGACUAGAUCCACAUUCAUCCA | 22 | mir-279 |
| tci-miR-279-5p | AUGAAUGCGUUUCUAGACCAUG | 22 | mir-279 |
| tci-miR-281-3p | UGUCAUGGAGUUGCUCUCUUUC | 22 | mir-46 |
| tci-miR-281-5p | AAGAGAGCUUAUCCGUAGACAG | 22 | mir-46 |
| tci-miR-305-3p | GACAUCUGAAGAAGUACAUUUA | 22 |  |
| tci-miR-305-5p | UUUGUACUUCAUCAGGUGCUCUG | 23 |  |
| tci-miR-307-3p | UCACAACCUCCUUGAGUGAGUGA | 23 | mir-67 |
| tci-miR-317-3p | UGAACACAGCUGGUGGUAUCUCAG | 24 | mir-317 |
| tci-miR-317-5p | UGGGUACCACGCUGGGCUCACA | 22 | mir-317 |
| tci-miR-34-3p | CCACUGAUUGCACUGCCCAUA | 21 | mir-34 |
| tci-miR-34-5p | UGGCAGUGUGGUUAGCUGGUU | 21 | mir-34 |
| tci-miR-3931-3p | UACUUUGAGUCGGUACGAAUCA | 22 | mir-3931 |
| tci-miR-3931-5p | AUUUGUACCGAUUCAUUGAUA | 21 | mir-3931 |
| tci-miR-5727-3p | GCCGCUCUUAUGAUUGCACGA | 21 | mir-5727 |
| tci-miR-5728-1-5p | ACAGCCGUAUGAGUGGCAGAAUA | 23 | mir-5728 |
| tci-miR-5728-3p | UUUGCCACUCAUACGGAUGCU | 21 | mir-5728 |
| tci-miR-5729a-5p | ACCCUGACUUGUGGUAAGAUAAGC | 24 | mir-5729 |
| tci-miR-5729b-3p | GAUCGAACCCGGUCAGGGUGC | 21 | mir-5729 |
| tci-miR-5729b-5p | ACCCUGACCAGUGGUAAGAUAAGC | 24 | mir-5729 |
| tci-miR-5731-3p | UGGGCACCUAGGCUAUGUCCA | 21 |  |
| tci-miR-5732-3p | UGCCACAGCUAUGACUGUACC | 21 | mir-5727 |
| tci-miR-5732-5p | AUACAGCCAUAGCUGUGGCAA | 21 | mir-5727 |
| tci-miR-5735-3p | UGGACAACAGGAUAAUGGCGU | 21 |  |
| tci-miR-5735-5p | ACCUUUCUCUUGUUGUCCACUG | 22 |  |
| tci-miR-5737-3p | CGGAUUCUCGAUUGGGGUUUU | 21 |  |
| tci-miR-5737-5p | UAAUCUCAACCGAUGAAUCCGAG | 23 |  |
| tci-miR-71-3p | UCUCACUACUUUGUCUUUGGCG | 22 | mir-71 |
| tci-miR-71-5p | UGAAAGACAUGGGUAGUGAGAUG | 23 | mir-71 |
| tci-miR-7-3p | ACAAGAAAUCGCUAGCUUCCGA | 22 | mir-7 |
| tci-miR-745-3p | CAGCUGCCCAGUGAAGGGCUG | 21 |  |
| tci-miR-745-5p | AGUCCGUCUCUGGGCUGCAAGGC | 23 |  |
| tci-miR-7-5p | UGGAAGACUAGUGAUUUUGUUGUU | 24 | mir-7 |
| tci-miR-87-3p | UUGAGCAAAGUUUCAGGUGUGU | 22 | mir-87 |
| tci-miR-87-5p | AAGCCGGAACCUUUGACUCAAC | 22 | mir-87 |
| tci-miR-92-3p | UAUUGCACUCGUCCCGGCCUGU | 22 | mir-25 |
| tci-miR-92-5p | AGGCCUAGACUGGUGUCAAUAUU | 23 | mir-25 |
| tci-miR-9-3p | AUAAAGCUAUUUAACCAAAUUUG | 23 | mir-9 |
| tci-miR-9-5p | UCUUUGGUUAUUUAGCUUUCUGA | 23 | mir-9 |
| tci-miR-993a-3p | GAAGCUCGUUUCUACAGGUUUC | 22 | mir-993 |
| tci-miR-993a-5p | UACCCUGUAGACCCGGGCUUUUG | 23 | mir-993 |
| tci-miR-993b-3p | GAAGCUCGUUUUUACAGGUUUUC | 23 | mir-993 |
| tci-miR-993b-5p | UACCCUGUGAACCCGGGCUUUUG | 23 | mir-993 |
