# Supplementary material for: Identification of Differentially Expressed microRNAs between the Fenpropathrin Resistant and Susceptible Strains in Tetranychus cinnabarinus
Source: PLoS One. 2016 Apr 6;11(4):e0152924. doi: 10.1371/journal.pone.0152924 (PMC4822788; doi:10.1371/journal.pone.0152924)
Supplement: S8 Table — (DOCX) [file pone.0152924.s010.docx]

**S8 Table. The expression level comparison results of the known and novel miRNAs in TS and TR strains**

| **MiRNA name** | **TR1.tpm** | **TR2.tpm** | **TS1.tpm** | **TS2.tpm** | **Log_2_(TR/TS)** | **P value** | **Padj** | **Sig-lable** |
| --- | --- | --- | --- | --- | --- | --- | --- | --- |
| tci-miR-281-5p | 4574.014 | 4191.876 | 11302.02 | 11682.56 | -1.39 | 2.0388E-34 | 2.8339E-32 | * |
| tci-miR-281-3p | 19926.5 | 19367.15 | 40954.83 | 47291.52 | -1.16 | 6.8406E-29 | 4.7542E-27 | * |
| tci-miR-92-3p | 167.729 | 163.634 | 526.7581 | 1218.569 | -2.35 | 4.6239E-12 | 2.1424E-10 | * |
| novel_43 | 128.4411 | 102.4491 | 218.0533 | 1480.314 | -2.79 | 2.5013E-08 | 6.9537E-07 | * |
| novel_29 | 0 | 0 | 29.4005 | 20.358 | -6.75 | 6.5185E-07 | 0.000015101 | * |
| novel_68 | 22.6661 | 24.1894 | 71.0511 | 75.6152 | -1.63 | 8.1829E-07 | 0.000016249 | * |
| novel_50 | 34.7546 | 34.1497 | 80.8512 | 69.7987 | -1.13 | 0.0002461 | 0.0028507 | * |
| tci-miR-745-5p | 104.2639 | 132.3301 | 274.4042 | 253.0202 | -1.15 | 0.00031255 | 0.0033419 | * |
| novel_39 | 83.1089 | 86.7972 | 19.6003 | 49.4407 | 1.35 | 0.000017974 | 0.0002776 | * |
| novel_52 | 15.1107 | 18.4978 | 0 | 2.9083 | 3.45 | 0.00040599 | 0.0040309 | * |
| novel_47 | 119.3747 | 136.5988 | 63.701 | 40.7159 | 1.27 | 0.00069823 | 0.0064703 | * |
| novel_59 | 10.5775 | 14.229 | 0 | 0 | 5.75 | 0.0027089 | 0.022149 | * |
| tci-miR-210-3p | 3476.976 | 4241.677 | 2430.437 | 2251.008 | 0.72 | 9.9906E-09 | 3.4717E-07 |  |
| tci-miR-12a-3p | 10686.3 | 10075.58 | 13845.16 | 15373.16 | -0.49 | 3.9267E-06 | 0.000068226 |  |
| tci-miR-278-3p | 843.178 | 876.509 | 1862.029 | 1544.296 | -1.00 | 0.000054128 | 0.00075238 |  |
| novel_5 | 819.0008 | 903.5442 | 1374.471 | 2070.694 | -0.97 | 0.00011032 | 0.0013941 |  |
| tci-miR-87-3p | 6913.153 | 6791.522 | 5375.383 | 5473.381 | 0.34 | 0.0020342 | 0.017672 |  |
| tci-miR-317-3p | 14202.56 | 15001.68 | 12664.24 | 10836.25 | 0.31 | 0.0042688 | 0.032965 |  |
| tci-miR-305-5p | 7771.441 | 9038.288 | 10145.61 | 10996.2 | -0.32 | 0.0047487 | 0.03474 |  |
| novel_18 | 24.1771 | 29.881 | 49.0008 | 58.1656 | -0.97 | 0.0064554 | 0.044865 |  |
| novel_53 | 1.5111 | 1.4229 | 12.2502 | 11.6331 | -2.84 | 0.0091089 | 0.060293 |  |
| tci-miR-124-1-5p | 466.9211 | 446.7919 | 298.9046 | 247.2037 | 0.73 | 0.010018 | 0.063294 |  |
| tci-miR-5729a-5p | 21.155 | 24.1894 | 36.7506 | 52.349 | -0.94 | 0.012708 | 0.076798 |  |
| novel_10 | 105.775 | 147.982 | 75.9512 | 63.9821 | 0.85 | 0.018936 | 0.10967 |  |
| novel_11 | 18.1329 | 31.3039 | 9.8002 | 8.7248 | 1.40 | 0.020237 | 0.11252 |  |
| tci-miR-190-5p | 10399.19 | 10799.84 | 9008.788 | 8727.744 | 0.26 | 0.021157 | 0.11311 |  |
| novel_40 | 1.5111 | 5.6916 | 2.45 | 31.9911 | -2.05 | 0.023945 | 0.12327 |  |
| novel_21 | 915.7094 | 870.8174 | 1553.324 | 1105.146 | -0.60 | 0.025646 | 0.12406 |  |
| novel_45 | 352140.1 | 349039.8 | 323811.7 | 272656.9 | 0.22 | 0.025882 | 0.12406 |  |
| tci-miR-993b-3p | 12.0886 | 8.5374 | 0 | 2.9083 | 2.74 | 0.03176 | 0.14715 |  |
| tci-miR-1-3p | 66540.04 | 63353.39 | 75228.4 | 74062.23 | -0.20 | 0.035559 | 0.15944 |  |
| tci-miR-263a-5p | 45681.21 | 45003.61 | 46854.52 | 56848.12 | -0.18 | 0.058146 | 0.25257 |  |
| tci-miR-184-3p | 34763.71 | 36964.21 | 37296.92 | 44644.99 | -0.18 | 0.061059 | 0.25719 |  |
| tci-miR-10-3p | 2630.776 | 2601.069 | 3214.449 | 2876.288 | -0.23 | 0.06343 | 0.25932 |  |
| novel_2 | 1699.956 | 1698.948 | 1313.22 | 1206.936 | 0.43 | 0.068936 | 0.25964 |  |
| novel_65 | 9.0664 | 11.3832 | 2.45 | 2.9083 | 1.86 | 0.069112 | 0.25964 |  |
| tci-miR-124-2-5p | 327.9025 | 297.387 | 218.0533 | 209.3961 | 0.54 | 0.068462 | 0.25964 |  |
| tci-miR-137-5p | 4.5332 | 5.6916 | 9.8002 | 20.358 | -1.47 | 0.076957 | 0.2815 |  |
| novel_55 | 51.3764 | 54.0704 | 34.3005 | 34.8993 | 0.61 | 0.082746 | 0.29491 |  |
| novel_14 | 34.7546 | 52.6475 | 31.8505 | 23.2662 | 0.65 | 0.1034 | 0.3558 |  |
| tci-miR-12a-5p | 1118.193 | 1033.029 | 1335.271 | 1494.855 | -0.39 | 0.10495 | 0.3558 |  |
| novel_46 | 12.0886 | 31.3039 | 9.8002 | 11.6331 | 1.03 | 0.11158 | 0.36369 |  |
| tci-miR-124-3p | 1240.59 | 1350.336 | 972.6649 | 1006.264 | 0.39 | 0.11251 | 0.36369 |  |
| tci-miR-5737-3p | 95.1975 | 91.0659 | 75.9512 | 63.9821 | 0.40 | 0.11772 | 0.37188 |  |
| novel_41 | 1570.003 | 1488.358 | 1619.475 | 2341.164 | -0.35 | 0.13064 | 0.39475 |  |
| tci-miR-1-5p | 2316.473 | 2094.515 | 2947.395 | 2239.375 | -0.25 | 0.12863 | 0.39475 |  |
| novel_26 | 0 | 0 | 4.9001 | 2.9083 | -4.16 | 0.13637 | 0.40095 |  |
| novel_57 | 6.0443 | 8.5374 | 0 | 2.9083 | 2.27 | 0.13846 | 0.40095 |  |
| tci-miR-10-5p | 4676.767 | 4667.126 | 5110.779 | 5354.141 | -0.16 | 0.14538 | 0.40416 |  |
| tci-miR-12b-3p | 1190.724 | 1220.852 | 967.7649 | 910.2912 | 0.36 | 0.14379 | 0.40416 |  |
| novel_42 | 528.8751 | 599.0427 | 431.2066 | 436.2418 | 0.38 | 0.15936 | 0.43432 |  |
| novel_56 | 22.6661 | 21.3436 | 9.8002 | 17.4497 | 0.72 | 0.18833 | 0.4946 |  |
| tci-miR-190-3p | 52.8875 | 61.1849 | 63.701 | 87.2484 | -0.38 | 0.18859 | 0.4946 |  |
| tci-miR-87-5p | 51.3764 | 65.4536 | 51.4508 | 34.8993 | 0.42 | 0.19246 | 0.49541 |  |
| novel_17 | 27.1993 | 42.6871 | 29.4005 | 17.4497 | 0.55 | 0.20405 | 0.51553 |  |
| tci-miR-34-5p | 16100.47 | 15407.21 | 16290.3 | 18371.6 | -0.13 | 0.2077 | 0.51553 |  |
| tci-miR-133-3p | 6831.555 | 7312.305 | 6722.903 | 6084.119 | 0.14 | 0.21787 | 0.53129 |  |
| novel_13 | 33.2436 | 19.9207 | 22.0503 | 11.6331 | 0.60 | 0.23345 | 0.55 |  |
| tci-miR-210-5p | 21.155 | 31.3039 | 19.6003 | 14.5414 | 0.60 | 0.23345 | 0.55 |  |
| tci-miR-279-3p | 6183.305 | 6361.805 | 7195.761 | 6523.269 | -0.13 | 0.25738 | 0.59144 |  |
| tci-miR-307-3p | 867.3551 | 772.637 | 715.411 | 625.2799 | 0.28 | 0.25955 | 0.59144 |  |
| tci-miR-278-5p | 132.9743 | 133.753 | 166.6026 | 177.405 | -0.36 | 0.26796 | 0.59552 |  |
| tci-miR-5729b-3p | 0 | 0 | 7.3501 | 0 | -4.16 | 0.26991 | 0.59552 |  |
| novel_12 | 34.7546 | 49.8016 | 31.8505 | 29.0828 | 0.47 | 0.28191 | 0.60637 |  |
| novel_34 | 27.1993 | 35.5726 | 29.4005 | 14.5414 | 0.47 | 0.28792 | 0.60637 |  |
| tci-miR-3931-3p | 11942 | 11428.77 | 10395.51 | 11121.26 | 0.12 | 0.28632 | 0.60637 |  |
| tci-miR-263b-5p | 42007.79 | 42877.8 | 42929.56 | 48181.45 | -0.09 | 0.29568 | 0.61342 |  |
| novel_62 | 36.2657 | 27.0352 | 44.1007 | 37.8076 | -0.39 | 0.31351 | 0.64085 |  |
| novel_44 | 25.6882 | 17.0749 | 14.7002 | 14.5414 | 0.53 | 0.32466 | 0.64608 |  |
| novel_6 | 1.5111 | 2.8458 | 9.8002 | 2.9083 | -1.50 | 0.32536 | 0.64608 |  |
| novel_37 | 63.465 | 61.1849 | 51.4508 | 52.349 | 0.26 | 0.36042 | 0.7056 |  |
| novel_4 | 1349.387 | 1387.332 | 1715.026 | 1462.864 | -0.22 | 0.36755 | 0.70957 |  |
| novel_31 | 492.6093 | 594.774 | 700.7108 | 572.9309 | -0.24 | 0.3949 | 0.74037 |  |
| novel_33 | 63.465 | 56.9162 | 56.3509 | 87.2484 | -0.23 | 0.41095 | 0.74037 |  |
| novel_54 | 6.0443 | 2.8458 | 7.3501 | 8.7248 | -0.83 | 0.41545 | 0.74037 |  |
| novel_67 | 10.5775 | 5.6916 | 2.45 | 5.8166 | 0.97 | 0.41725 | 0.74037 |  |
| tci-miR-137-3p | 2778.861 | 2638.064 | 2704.842 | 3158.391 | -0.11 | 0.42573 | 0.74037 |  |
| tci-miR-34-3p | 332.4358 | 369.9551 | 477.7573 | 346.0852 | -0.25 | 0.42352 | 0.74037 |  |
| tci-miR-71-3p | 13938.12 | 13879.01 | 13119.95 | 13142.51 | 0.08 | 0.42611 | 0.74037 |  |
| tci-miR-745-3p | 108.7972 | 116.6781 | 117.6018 | 159.9553 | -0.28 | 0.40124 | 0.74037 |  |
| novel_51 | 93.6864 | 95.3346 | 68.6011 | 95.9732 | 0.22 | 0.45599 | 0.78183 |  |
| tci-miR-5735-3p | 107716.7 | 102018 | 86961.63 | 111203.9 | 0.10 | 0.46122 | 0.78183 |  |
| novel_25 | 7.5554 | 2.8458 | 0 | 2.9083 | 1.78 | 0.49019 | 0.80091 |  |
| novel_3 | 2337.628 | 2195.541 | 2224.634 | 2629.084 | -0.09 | 0.51438 | 0.80091 |  |
| novel_35 | 9.0664 | 11.3832 | 4.9001 | 8.7248 | 0.61 | 0.49018 | 0.80091 |  |
| novel_48 | 15.1107 | 7.1145 | 7.3501 | 5.8166 | 0.71 | 0.49229 | 0.80091 |  |
| novel_64 | 4.5332 | 7.1145 | 2.45 | 2.9083 | 1.07 | 0.53599 | 0.80091 |  |
| novel_69 | 4.5332 | 15.6519 | 12.2502 | 0 | 0.61 | 0.50195 | 0.80091 |  |
| novel_8 | 281.0593 | 308.7702 | 352.8054 | 319.9107 | -0.19 | 0.52124 | 0.80091 |  |
| tci-miR-252-5p | 809.9344 | 883.6235 | 957.9647 | 919.0161 | -0.15 | 0.55643 | 0.80091 |  |
| tci-miR-2-5p | 80.0868 | 116.6781 | 120.0518 | 107.6063 | -0.21 | 0.55796 | 0.80091 |  |
| tci-miR-276-3p | 26480.02 | 25677.73 | 25073.68 | 29361.98 | -0.05 | 0.55752 | 0.80091 |  |
| tci-miR-305-3p | 132.9743 | 112.4094 | 137.2021 | 69.7987 | 0.20 | 0.55891 | 0.80091 |  |
| tci-miR-3931-5p | 6.0443 | 5.6916 | 2.45 | 2.9083 | 1.07 | 0.5274 | 0.80091 |  |
| tci-miR-5731-3p | 3.0221 | 0 | 0 | 0 | 2.85 | 0.50004 | 0.80091 |  |
| tci-miR-5735-5p | 175.2843 | 206.3211 | 254.8039 | 183.2216 | -0.22 | 0.54287 | 0.80091 |  |
| tci-miR-7-3p | 52.8875 | 75.4139 | 61.2509 | 87.2484 | -0.18 | 0.52605 | 0.80091 |  |
| tci-miR-2-3p | 6654.759 | 7548.507 | 6335.797 | 7238.706 | 0.08 | 0.58901 | 0.83226 |  |
| tci-miR-5728-3p | 956.5083 | 848.0509 | 857.5132 | 782.327 | 0.13 | 0.59276 | 0.83226 |  |
| novel_1 | 59244.59 | 62397.2 | 64195.89 | 53337.83 | 0.04 | 0.63185 | 0.87613 |  |
| tci-miR-252-3p | 28.7104 | 35.5726 | 44.1007 | 29.0828 | -0.21 | 0.63661 | 0.87613 |  |
| tci-miR-5729b-5p | 64.9761 | 83.9513 | 73.5011 | 90.1566 | -0.12 | 0.6504 | 0.88633 |  |
| tci-miR-9-3p | 726.8255 | 738.4873 | 676.2104 | 904.4747 | -0.09 | 0.67958 | 0.9171 |  |
| novel_28 | 37.7768 | 25.6123 | 31.8505 | 23.2662 | 0.17 | 0.6995 | 0.93491 |  |
| novel_9 | 374.7458 | 308.7702 | 330.7551 | 305.3693 | 0.09 | 0.72969 | 0.93704 |  |
| tci-miR-276-5p | 64.9761 | 58.3391 | 66.151 | 66.8904 | -0.11 | 0.72608 | 0.93704 |  |
| tci-miR-5732-3p | 126.93 | 108.1407 | 117.6018 | 139.5974 | -0.12 | 0.7348 | 0.93704 |  |
| tci-miR-5737-5p | 1740.755 | 1745.904 | 1648.875 | 1634.453 | 0.09 | 0.7131 | 0.93704 |  |
| tci-miR-993a-5p | 4395.707 | 4719.773 | 4512.969 | 4865.55 | -0.03 | 0.72342 | 0.93704 |  |
| novel_70 | 3.0221 | 7.1145 | 2.45 | 2.9083 | 0.89 | 0.74389 | 0.94 |  |
| novel_66 | 7.5554 | 2.8458 | 4.9001 | 0 | 0.89 | 0.75167 | 0.94128 |  |
| novel_30 | 137.5075 | 176.4401 | 144.5522 | 148.3222 | 0.11 | 0.77292 | 0.94242 |  |
| novel_49 | 27.1993 | 21.3436 | 22.0503 | 20.358 | 0.18 | 0.77125 | 0.94242 |  |
| tci-miR-9-5p | 44516.17 | 48165.31 | 45908.8 | 45011.43 | 0.03 | 0.76807 | 0.94242 |  |
| tci-miR-7-5p | 32225.11 | 34095.63 | 33504.26 | 34015.23 | -0.02 | 0.78557 | 0.94951 |  |
| novel_36 | 7.5554 | 7.1145 | 14.7002 | 2.9083 | -0.34 | 0.81777 | 0.96658 |  |
| novel_7 | 566.6519 | 507.9768 | 531.6582 | 497.3157 | 0.06 | 0.82055 | 0.96658 |  |
| tci-miR-5732-5p | 9.0664 | 5.6916 | 14.7002 | 2.9083 | -0.34 | 0.81748 | 0.96658 |  |
| tci-miR-12b-5p | 492.6093 | 506.5539 | 490.0075 | 468.2329 | 0.06 | 0.83807 | 0.97106 |  |
| tci-miR-993b-5p | 160.1736 | 149.4049 | 149.4523 | 145.4139 | 0.07 | 0.83832 | 0.97106 |  |
| novel_32 | 15.1107 | 14.229 | 19.6003 | 14.5414 | -0.23 | 0.86021 | 0.98794 |  |
| novel_61 | 151.1072 | 109.5636 | 110.2517 | 139.5974 | 0.07 | 0.87034 | 0.98794 |  |
| tci-miR-71-5p | 5965.711 | 5701.577 | 5439.084 | 6081.211 | 0.02 | 0.87422 | 0.98794 |  |
| novel_20 | 6.0443 | 9.9603 | 12.2502 | 5.8166 | -0.20 | 1 | 1 |  |
| novel_22 | 37.7768 | 27.0352 | 29.4005 | 37.8076 | -0.04 | 1 | 1 |  |
| novel_27 | 6.0443 | 4.2687 | 7.3501 | 2.9083 | -0.05 | 1 | 1 |  |
| novel_58 | 4.5332 | 1.4229 | 2.45 | 2.9083 | 0.13 | 1 | 1 |  |
| novel_63 | 1.5111 | 1.4229 | 0 | 2.9083 | 0.12 | 1 | 1 |  |
| novel_71 | 3.0221 | 2.8458 | 2.45 | 2.9083 | 0.13 | 1 | 1 |  |
| tci-miR-133-5p | 1.5111 | 0 | 0 | 0 | 2.04 | 1 | 1 |  |
| tci-miR-184-5p | 175.2843 | 182.1317 | 188.6529 | 177.405 | -0.04 | 0.93918 | 1 |  |
| tci-miR-263a-3p | 149.5961 | 159.3653 | 147.0023 | 157.0471 | 0.03 | 0.97679 | 1 |  |
| tci-miR-263b-3p | 9.0664 | 11.3832 | 2.45 | 17.4497 | 0.14 | 1 | 1 |  |
| tci-miR-279-5p | 25.6882 | 27.0352 | 34.3005 | 20.358 | -0.08 | 1 | 1 |  |
| tci-miR-317-5p | 188.884 | 182.1317 | 208.2532 | 162.8636 | -0.02 | 1 | 1 |  |
| tci-miR-5727-3p | 1.5111 | 0 | 0 | 0 | 2.04 | 1 | 1 |  |
| tci-miR-5728-1-5p | 0 | 1.4229 | 0 | 0 | 2.04 | 1 | 1 |  |
| tci-miR-92-5p | 0 | 0 | 2.45 | 0 | -2.73 | 1 | 1 |  |
| tci-miR-993a-3p | 430.6554 | 546.3952 | 394.4561 | 587.4723 | 0.02 | 0.99197 | 1 |  |

Sig-label: * represents |log2 (fold change)| > 1 and adjusted padj<0.05
